# Treatment of Dysmenorrhœa with the Pessary

**Published:** 1840-06

**Authors:** 


### TREATMENT OF DYSMENORRHŒA WITH THE PESSARY.

Our friend, Dr. Mosby, of this city, has been in the habit for many years, of treating dysmenorrhœa, by the introduction, on the approach of the catamenial period, of a pessary, carried high into the vagina. He supposes, that in these cases, the uterus is engorged, and at the same time is drawn or pressed down into the pelvis, where it is compressed and in some degree strangulated. He has seen the pain instantly relieved, by pushing up the organ and sustaining it by a pessary. His practice is to administer a cathartic previously to the attack. D.

